# Hypotonic Hyponatremia: Diagnosis and Management

**DOI:** 10.3390/jcm15208056

**Published:** 2026-10-16

**Authors:** Raymond E. Garrett, Brent Wagner, Ramin Sam, Mark L. Unruh, Susie Q. Lew, Christos P. Argyropoulos, Maria-Eleni Roumelioti, Deepak Malhotra, Mark Rohrscheib, Darlene Vigil, Todd S. Ing, Antonios H. Tzamaloukas

**Affiliations:** 1 Trauma Research, Swedish Medical Center, Englewood, CO 80113, USA; docrayconsults@me.com; 2 Division of Nephrology, Department of Medicine, University of New Mexico School of Medicine, Albuquerque, NM 87122, USAcargyropoulos@salud.unm.edu (C.P.A.); mroumelioti@salud.unm.edu (M.-E.R.); mrohrscheib@salud.unm.edu (M.R.); 3 Kidney Institute of New Mexico, University of New Mexico Health Sciences Center, Albuquerque, NM 87122, USA; 4 Raymond G. Murphy Veterans Affairs Medical Center, Albuquerque, NM 87108, USA; mlunruh@salud.unm.edu; 5 Division of Nephrology, Department of Medicine, Zuckerberg San Francisco General Hospital, University of California in San Francisco School of Medicine, San Francisco, CA 94110, USA; ramin.sam@ucsf.edu; 6 Department of Medicine, University of New Mexico School of Medicine, Albuquerque, NM 87122, USA; 7 Division of Renal Diseases and Hypertension, Department of Medicine, The George Washington University School of Medicine and Health Sciences, Washington, DC 20037, USA; sqlew@gwu.edu; 8 Division of Nephrology, Department of Medicine, University of Toledo School of Medicine, Toledo, OH 43614, USA; deepak.malhotra@utoledo.edu; 9 Division of Nephrology, Department of Medicine, Raymond G. Murphy Veterans Affairs Medical Center, University of New Mexico School of Medicine, Albuquerque, NM 87108, USA; darlene.vigil@va.gov; 10 Department of Medicine, Stritch School of Medicine, Loyola University Chicago, Maywood, IL 60153, USA

**Keywords:** hypotonic hyponatremia, hypovolemia, euvolemia, hypervolemia, acute hyponatremia, chronic hyponatremia, osmotic diuresis, vaptans, vasopressin infusion, saline infusion, osmotic demyelination syndrome

## Abstract

The diagnosis and treatment of hyponatremia present challenges to clinicians and opportunities to improve our understanding of body water, sodium and potassium homeostasis and disorders. The first step in the diagnosis of every case of hyponatremia is to categorize it as hypotonic hyponatremia, hypertonic hyponatremia, or pseudohyponatremia. The diagnostic steps that follow the verification of hypotonic hyponatremia include: (a) the identification of the medical condition that caused the hyponatremia; (b) the evaluation of the mechanism of hyponatremia, including the computation of the magnitude of the changes in body water, sodium, and potassium that were responsible for the hyponatremia. Whether the extracellular volume is low, normal, or high provides key information for the determination of the mechanism of hyponatremia and the choice of its treatment method; and (c) the determination of the duration of hyponatremia. Whether hyponatremia is acute or chronic (i.e., lasting more than 48 h) has important implications for its manifestations and management. This perspective reviews the diagnostic categories and various aspects of treatment strategies of hypotonic hyponatremia, including choosing the method(s) for increasing the electrolyte-free renal water clearance, decreasing this clearance during treatment to prevent excessively rapid correction of hyponatremia, determining the type and amount of saline infused for correction of the hyponatremia (choice of isotonic versus hypertonic saline and of the target rise in serum sodium, which determines, along with other parameters, the volume of saline infused), and dialytic methods for correcting hyponatremia. The aim of this perspective is to identify topics that require further research, including diagnostic topics and treatment areas.

## 1. Introduction

For the body cells to function normally, preservation of their volume is essential. Tonicity of a solution refers to its property to change the volume of cells suspended in it by osmotic water transfer across the cell membranes. Disorders of serum tonicity constitute the major cause of intracellular volume abnormalities. Table 1 outlines the six parameters required for determining the cell volume [1].

Note that the serum has two volume components, the serum water content (*SWC*) and the serum solid content (*SSC*), which is composed of proteins and lipids. The relationship between these two components is given as *SWC* + *SSC* = 1.0 (normally, *SWC* = 0.93 and *SSC* = 0.07 of serum volume) [1]. Only the *SWC* contains the dissolved solutes. The numerical differences between *[Na^+^]_SW_* and *[Na^+^]_S_* and between *[Osm]_LS_* and *[Osm]_RS_* are a consequence of the higher concentrations of dissolved solutes in serum water than in serum. The relationships between the concentrations in serum and serum water are expressed as *[Na^+^]_S_* = *[Na^+^]_SW_* × *SWC*; and *[Osm]_RS_* = *[Osm]_LS_* × *SWC* [1], when *[Osm]_RS_* is computed by an ideal formula. For example, at *SWC* = 0.93 and *[Na^+^]_SW_* = 150 mmol/L, *[Na^+^]_S_* = 150 × 0.93 = 139.5 mmol/L.

The main clinical application of *[Na^+^]_S_* is as an index of serum tonicity [1,2,3]. Hyponatremia is defined as *[Na^+^]_S_* < 135 mmol/L in guidelines of its treatment [4,5] and in studies analyzing the clinical impact of *[Na^+^]_S_* [6,7,8]. However, the normal range for *[Na^+^]_S_*, typically reported as 135–145 mmol/L, is wide. Hyponatremia manifestations were observed even at decreases of *[Na^+^]_S_* within this normal range [7]. The normal range of *[Na^+^]_S_* in United States adults was computed as 138–142 mmol/L [9]. The reported cut-off values of *[Na^+^]_S_* for hyponatremia in clinical studies range from <130 mmol/L [10] to <138 mmol/L [11].

There are three pathophysiologic categories of hyponatremia based on tonicity: hypotonic hyponatremia, hypertonic hyponatremia, and pseudohyponatremia or isotonic hyponatremia [1,12,13,14]. In hypotonic hyponatremia, cell swelling results from a relative excess of body water over osmotically effective body solutes. Hypertonic hyponatremia results from osmotic water exit from cells driven by an excess of extracellular solutes other than sodium salts and causing cell shrinkage and dilution of extracellular solutes, including sodium. Pseudohyponatremia indicates a falsely low estimate of *[Na^+^]_SW_* resulting from a reported low *[Na^+^]_S_*. In pure pseudohyponatremia, cell volume remains within the normal range.

Hypotonic hyponatremia is the most common electrolyte abnormality observed in many patient categories [15,16,17,18,19,20,21,22,23,24]. It has significant morbidity and mortality [16,24,25,26,27,28,29,30,31,32,33,34,35,36]. Several reports have addressed its mechanisms [25,36,37,38,39,40,41,42,43,44,45,46,47,48,49] and treatment methods [4,5,29,30,31,32,33,34,35,36,37,38,39,40,41,42,43,44,45,46,47,48,49,50,51,52,53,54,55]. Its diagnostic categories and management methods have recently been debated. This perspective addresses the steps of diagnosis and the methods of treatment of hypotonic hyponatremia. Emphasis is placed on currently disputed issues.

## 2. Diagnosis of Hypotonic Hyponatremia

### 2.1. Differentiating the Tonicity Category of Hyponatremia

The first step in the diagnosis of hyponatremia is to determine its tonicity category [1,4,5,12,13,14,19]. The identification of the category of pure forms of hyponatremia, following the reporting of a low *[Na^+^]_S_*, consists of the following steps [1]: (a) Measurement of *[Osm]_LS_*. (b) Computation of *[Osm]_RS_* using several formulas [56], with the formula in Table 1 the most commonly used [57,58]. (c) Computation of the *[Gap]_OsmS_* [59,60,61]. The normal range of the *[Gap]_OsmS_* using the formula in Table 1 to calculate *[Osm]_RS_* is up to 10 mOsm/L [59]. (d) Computation of the *[Ton]_S_*. In hypertonic hyponatremia, *[Ton]_S_* calculated by the formula in Table 1 is specific for hyperglycemia. This formula systematically underestimates the actual serum tonicity in hypertonic hyponatremia caused by the gain of exogenous extracellular solutes, other than sodium salts [1]. (e) Measurement or computation of the *[Na]_SW_* [2,62]. Table 2 shows the characteristic features of the pure categories of hyponatremia without any other changes in body solutes or water [1].

A case of hyponatremia may represent a pure form, or it may be combined with other types of dysnatremia or with disturbances in body fluids of solutes not affecting the *[Na^+^]_S_* and the tonicity state. Examples of applying the parameters in Table 2 in the classification of both pure hyponatremia and its combination with other solute abnormalities have been reported [44,62,63,64].

Abnormally high values of the *[Gap]_OsmS_* either establish the diagnosis of pseudohyponatremia or indicate high serum levels of endogenous (e.g., in the sick cell syndrome) [1] or exogenous solutes not included in the formulas computing the *[Osm]_RS_* [59,60,61]. Among solutes distributed in body water that do not affect the cell volume in the steady state but contribute to *[Osm]_LS_*, only urea is included in the formulas calculating *[Osm]_RS_*. High *[Urea]_S_* levels do not cause high *[Gap]_OsmS_* values. All exogenous solutes distributed in the body water cause high *[Gap]_OsmS_* values by contributing to *[Osm]_LS_* but not to *[Osm]_RS_*. Exogenous solutes with extracellular distribution cause also a high *[Gap]_OsnS_*. Identification of the mechanism of a high *[Gap]_OsmS_* is critical for differentiating between pseudohyponatremia, hypertonic hyponatremia from excess of exogenous extracellular solutes, and hypotonic hyponatremia with an excess of exogenous solutes distributed in body water.

Hyponatremia, combined with other solute and fluid abnormalities, affects several parameters of Table 2 [1,14,18,20,65]. For example, low levels of *[Na^+^]_S_*, *[Ton]_S_*, and *[Na^+^]_SW_* are observed in all cases of hypotonic hyponatremia. However, hypotonic hyponatremia is also encountered in patients with elevated levels of endogenous (urea) or exogenous (e.g., ethanol) solutes distributed in body water. In azotemic hypotonic hyponatremia, *[Osm]_LS_* and *[Osm]_RS_* may be low, normal, or high, depending on the levels of *[Na^+^]_S_* and *[Urea]_S_*, and *[Gap]_OsmS_* is normal or slightly elevated [66].

Alcohol dependence is often associated with hypotonic hyponatremia [65]. Patients with no recent alcohol intake exhibit the features of pure hypotonic hyponatremia (Table 2). However, in patients with excessive blood levels of ethanol and hypotonic hyponatremia, *[Osm]_LS_* may be low, normal, or high, depending on the ethanol blood level, but *[Osm]_RS_* and *[Ton]_S_* are low because of the low *[Na]_S_*, and *[Gap]_OsmS_* is high, reflecting the serum ethanol level [60,61,67]. The same pattern of tonicity parameters is encountered in hypertonic hyponatremia caused by exogenous solutes with extracellular distribution, leading to misclassification and mistreatment [1]. Determining the state of tonicity requires identification of the exogenous solute by careful history detection or laboratory measurement. Cases combining all three tonicity categories of hyponatremia have been reported [68].

Pseudohyponatremia is a consequence of measuring *[Na^+^]_S_* by a method applying pre-measurement dilution of the serum sample, including flame photometry and indirect potentiometry [62]. Auto-analyzers measuring *[Na^+^]_S_* by indirect potentiometry compute low *[Na^+^]_S_* values that indirectly indicate falsely low *[Na^+^]_SW_* levels in sera with high *SSC* values due to hyperproteinemia or hyperlipidemia and corresponding low *SWC* values [62].

Pre-measurement dilution is not used in direct potentiometry, which provides *[Na^+^]_S_* values accurately reflecting *[Na^+^]_SW_* levels [62]. The *[Gap]_OsmS_* is high in sera with high *SSC* values when *[Na^+^]_S_* is measured by indirect potentiometry, because the *[Osm]_RS_* is low due to the low *[Na^+^]_S_* while the *[Osm]_LS_* is in the normal range due to the normal value of the *[Na^+^]_SW_* (Table 2). The *[Gap]_OsmS_* is normalized when *[Na^+^]_S_* is measured by direct potentiometry establishing the diagnosis of pseudohyponatremia. In addition to high *SSC*, pseudohyponatremia was reported in plasma samples of patients with non-Hodgkin’s lymphoma [69], or acute lymphoblastic leukemia [70], and in sera of patients with hereditary stomatocytosis [71]. Pseudohyponatremia would be eliminated by measuring, without pre-measurement dilution, and reporting *[Na^+^]_SW_* [62,72].

### 2.2. Determinants of [Na^+^]_SW_ and [Na^+^]_S_

A study by Edelman and coinvestigators provided the current approach to dysnatremias [73,74]. This study compared statistically *[Na^+^]_SW_*, total body exchangeable sodium (*TBNa_Exch_*), which represents the osmotically active extracellular solutes, total body exchangeable potassium (*TBK_Exch_*), which represents the osmotically active intracellular solutes, and total body water (*TBW*). *[Na^+^]_SW_* was estimated by a proper method from *[Na^+^]_S_* measured by flame photometry. *TBNa_Exch_*, *TBK_Exch_*, and *TBW* were measured by radio-isotopic methods. The study, which was performed in patients with *[Na^+^]_SW_* ranging from 108.9 to 192.5 mmol/L, developed a multiple linear regression formula expressing *[Na^+^]_SW_* as a function of *TBNa_Exch_*, *TBK_Exch_*, and *TBW* [73]. Based on the Edelman formula, Rose proposed a simpler formula expressing *[Na^+^]_S_* as a function of the same three determinants [75]. Table 3 shows the Edelman and Rose formulas.

Statistically, the 95% confidence interval (CI) of the regression coefficient of 1.11 in the Edelman formula includes the Rose coefficient 1.0, while the 95% CI of the −25.6 y-intercept in the Edelman formula, calculated as −5.2 to −46.0, does not include the Rose coefficient zero [76].

Current methods for diagnosing true dysnatremias (not pseudohyponatremia or pseudohypernatremia) aim to identify the direction and magnitude of changes in the three determinants of [Na^+^]_SW_ or [Na^+^]_S_ that alter the fraction in the Edelman or Rose formulas. The methods for correcting dysnatremias aim to normalize this fraction.

One limitation of the Edelman study is that it lacks data on patients studied when their *[Na^+^]_SW_* is changing [77]. The change in *[Na^+^]_SW_* during the development or correction of dysnatremias could be influenced by factors not included in this study. In the body, sodium exists in (a) the osmotically active compartment, mainly in extracellular fluids; (b) the exchangeable osmotically inactive compartment, which consists of anionic proteoglycans in skin, cartilage, bones, muscles, and endothelial surface layers [77], e.g., glycosaminoglycan [78]; and (c) the non-exchangeable osmotically inactive compartment. Exchanges of sodium between the osmotically active and the exchangeable osmotically inactive compartment during development or correction of dysnatremias would affect the size of the change in *[Na^+^]_SW_* [77,79].

Exchanges of sodium between these two compartments have been suggested by the magnitude of the changes in *[Na^+^]_S_* during treatment of the syndrome of inappropriate antidiuretic hormone (ADH) secretion (SIADH) [80] and during development of hyponatremia in intense exercise [81] or from use of thiazides [82]. In experimental studies, the changes in *[Na^+^]_S_* in healthy humans after infusion of hypertonic saline [83] or after ingestion of a standardized volume of water [84], and in dogs with low serum bicarbonate secondary to either metabolic acidosis or respiratory alkalosis after infusion of hypertonic sodium bicarbonate [85], have also suggested interactions between the two sodium compartments. These interactions can potentially be significant during development or correction of hypotonic hyponatremia.

Other factors not studied in the Edelman study include genetic and disease influences on the metabolism of heparan sulfate, an essential component of proteoglycans, exchange of potassium between its intracellular osmotically active and inactive compartments, and binding of water to hydrophilic compounds, which limits the water available for ionic solvation [77]. Also, a repeated linear regression analysis of the data in the Edelman study found significant effects of varying body weight, edema, and presenting *[Na^+^]_SW_* on the coefficients of the Edelman formula [86]. This finding suggests that these three clinical characteristics should be accounted for to improve the accuracy of dysnatremia formulas and strengthens the suggestion that the relationship depicted in Edelman’s formula is probably not linear [76].

**Figure 1:**
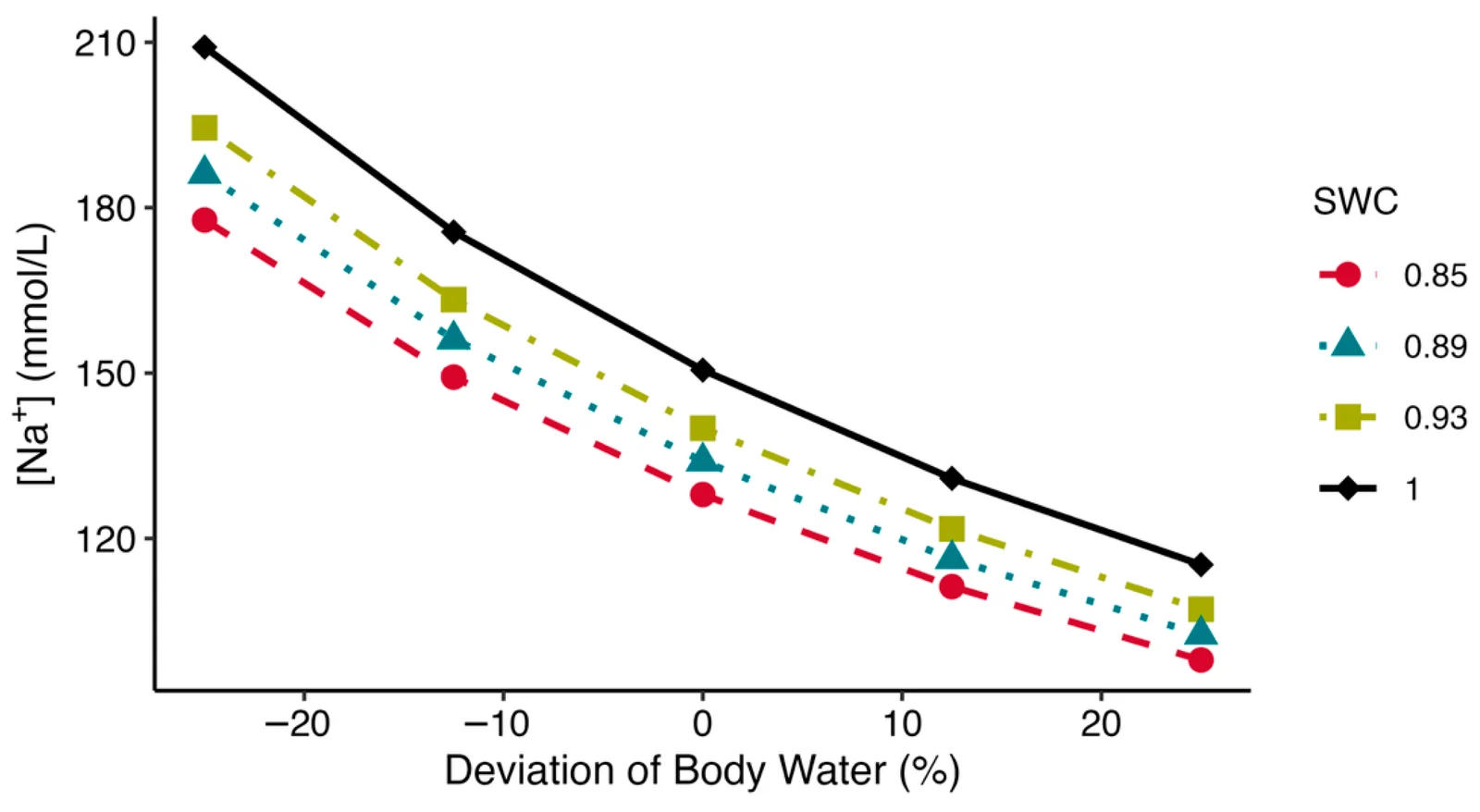
*[Na^+^]_SW_* values and *[Na^+^]_S_* values at different levels of both *TBW* and *SWC*.

Two crucial differences between the Edelman and Rose formulas should be noted. First, the Edelman formula expresses *[Na^+^]_SW_*, thereby eliminating pseudohyponatremia caused by high *SSC* and corresponding low *SWC* values. The Rose formula, which expresses *[Na^+^]_S_*, does not allow the distinction between hypotonic hyponatremia and pseudohyponatremia because multiple *[Na^+^]_S_* values, each corresponding to a specific *SWC*, can be found for the same *[Na^+^]_SW_* value. Figure 1 shows this relationship.

The figure shows *[Na^+^]_SW_* values (at *SWC* = 1.00) and the *[Na^+^]_S_* values corresponding to each *[Na^+^]_SW_* value at *SWC* values between 0.85 and 0.93 for *TBW* values between −20% and +20% of the normal value. Modification of the Rose formula using the mathematical relationship between *[Na^+^]_SW_* and *[Na^+^]_S_* (*[Na^+^]_S_* = *SWC* × (*TBNa_Exch_* + *TBK_Exch_)*/*TBW*) constitutes the essential step for the diagnosis of pseudohyponatremia using *[Na^+^]_S_* and *SWC*.

The second difference between the two formulas is that the Edelman formula may allow calculation of influences on the *[Na^+^]_SW_* by exchanges between the osmotically active and inactive sodium compartments. Such exchanges were proposed to be responsible for the −25.6 y-intercept of this formula [73,79]. The Rose formula does not contain any component that can be used to compute such exchanges.

*[Na^+^]_SW_* or *[Na^+^]_S_* computed by the Edelman and Rose formulas fails to yield accurate estimates of cell volume in hypertonic hyponatremia, e.g., in hyperglycemic crises. The serum parameters characterizing cell volume and the relationship between *TBW* and effective solutes in hyperglycemia have been investigated. The *[Ton]_S_* formula indicates the state of the cell volume [1]. However, the computed serum sodium after correction of the hyperglycemia without any changes in body water, sodium, and potassium (*[Na^+^]_Scor_*) indicates the relationship among *TBNa_Exch_*, *TBK_Exch_*, and *TBW*. *[Na^+^]_Scor_* is the proper parameter for calculating the tonicity of the replacement solutions [63,87,88]. Serum tonicity and *[Na^+^]_Scor_* should be monitored during treatment [63,87,88]. When *[Ton]_S_* is high, it should decrease by around 3 mOsm/L hourly [87]. Patients with oligo-anuria have negligible or no changes in body sodium, potassium, and water during the development or correction of hyperglycemia with insulin alone [89]. Studies in these patients [63,89] provided evidence supporting the computation of *[Na^+^]_Scor_* using Katz’s theoretical calculation of a rate of decrease in *[Na^+^]_S_* by 1.6 mmol/L for each 5.6 mmol/L (100 mg/dL) increase in *[Glucose]_S_* [90]. Note that in patients with hyperglycemia and marked hypervolemia, the observed rate of decrease in *[Na]_S_* was substantially lower than Katz’s rate [91], confirming theoretical calculations [92,93].

The correction of hypotonic hyponatremia by increasing the fraction (*TBNa_Exch_* + *TBK_Exch_*)/*TBW* could be improved by accounting for sodium exchange between the osmotically active and inactive compartments. *TBNa_Exch_* and *TBK_Exch_* may not be completely equivalent in determining *[Na^+^]_SW_* as the formulas suggest, and other cation or anion intake/output, such as magnesium salts, in large amounts could also affect the magnitude of the change in *[Na^+^]_S_* in hyponatremia. Experimental animal studies of the pathogenesis of dysnatremias and controlled animal and patient studies of the treatment of hypotonic hyponatremia addressing the limitations of the Edelman or Rose formulas, e.g., by measuring *TBW* and then infusing a volume of hypertonic saline computed to produce a specific rise in *[Na^+^]_S_* by one of the available formulas, are needed.

### 2.3. Specific Diagnostic Features of Hypotonic Hyponatremia

The steps in the diagnosis of hypotonic hyponatremia after ruling out hypertonic hyponatremia and pseudohyponatremia include the identification of (a) the medical condition responsible for the hyponatremia, (b) the mechanism(s) of hyponatremia, (c) the status of the extracellular volume (*ECV*), and (4) the duration of hyponatremia [13,51]. Careful evaluation of history and judicial use of laboratory tests are needed [4,5,36,37,38,39,40,41,42,43,44,94,95].

In terms of duration, hypotonic hyponatremia is classified as acute or chronic. The swelling of brain cells in this condition is reduced by rapid loss of sodium, potassium, chloride, and water from their intracellular compartment. A slower (up to 48 h) loss of organic osmolytes follows, including myoinositol, betaine, glutamine, γ-aminobutyric acid, taurine, and others [96]. After 48 h, hyponatremia is classified as chronic and is characterized by improvement in the symptoms of acute hyponatremia but causes other problems. These include alterations in neurological function, e.g., decreased synaptic release of excitatory neurotransmitters, which causes gait disorders, falls, lethargy, disorientation, headaches, nausea, and vomiting [96]. In addition, chronic hyponatremia increases the risk of neurological complications during correction of the *[Na^+^]_S_*.

The main mechanisms of hypotonic hyponatremia consist of (a) decreased renal excretion of water through inability to dilute the urine, or low solute intake causing low urinary water excretion, or substantially decreased renal function limiting water excretion; (b) excessive water intake; and (c) combinations of decreased excretion and increased intake of water [13,42,51]. Low water excretion, with high levels of urine osmolality (*[Osm]_LU_*) caused by high blood levels of ADH, accounts for a large fraction of hypotonic hyponatremias [13].

### 2.4. Extracellular Volume Status in Hypotonic Hyponatremias

Hyponatremia develops in patients with low, normal, or high extracellular volume (*ECV*) [4,5,13,51]. Sodium salts are the major solute determining *ECV*. In evaluating hypotonic hyponatremias, distinction between the states of *ECV* and of *TBW* that are not accompanied by proportional changes in effective body solutes [3,97,98,99] and detection of combinations of these states are critical. Hyponatremias with hypervolemia, hypovolemia, or euvolemia present diagnostic and treatment challenges [13].

Hypotonic hyponatremia with hypervolemia is typically encountered in congestive heart failure (CHF), nephrotic syndrome, hepatic cirrhosis [13,51], and during pregnancy or delivery [51,100]. In these disease categories, hyponatremia may also develop in patients with euvolemia or hypovolemia depending on salt and water intake, use of various categories of drugs, e.g., thiazides or renin angiotensin aldosterone system (RAAS) inhibitors [101], and extrarenal losses of salt and water. In the first three hypervolemic hyponatremias listed, urine sodium (*[Na^+^]_U_*) is low (<10 mmol/L) [13] and urine osmolality (*[Osm]_LU_*) is high because of ADH secretion stimulated by low effective arterial blood volume (*EABV*) [13,96,97,98,102]. *EABV* represents the blood volume responsible for organ perfusion. *ECV* and *EABV*, although intricately linked, are not expressions of the same entity. The *ECV* represents a major component of *EABV*. The other components include total red blood cell volume, cardiac output, arterial and venous vascular capacity, Starling forces between blood and interstitial fluids across capillary walls, endothelial barrier integrity, and gravity [99].

The terms hypovolemia, euvolemia, and hypervolemia refer to *ECV*, not to *EABV*. Unlike the *ECV*, *EABV* cannot be measured directly. Instead, a low *EABV* is inferred by manifestations occurring in hypovolemia, e.g., orthostatic hypotension and tachycardia, non-osmotic stimulation of ADH secretion, high serum levels of renin and aldosterone, low levels of *[Na^+^]_U_* and of fractional excretion of urea, and static and dynamic imaging (e.g., echocardiography, inferior vena cava ultrasonography) [103,104,105,106,107]. In cases of low *EABV*, therapy is guided primarily by *EABV* considerations, followed by optimization of the *ECV* “within reason”. Low cardiac output in CHF constitutes a representative state of low *EABV* in the setting of hypervolemia [99,108]. The low *[Na^+^]_U_* in CHF indicates that the kidneys see a low *EABV* even though the *ECV* is above its normal range, and other organs are probably similarly affected.

Hyponatremia in chronic kidney disease (CKD) has been classified as hypervolemic [5,51]. Despite having a high incidence of hypotonic hyponatremia, patients with CKD, who do not have a low *EABV*, are unable to concentrate the urine early during their condition, while their ability to dilute the urine remains intact for a longer period [18,20,109]. They also exhibit impaired ability to conserve sodium [110]. As CKD advances, the kidneys’ diluting ability is also affected. CKD patients with hyponatremia present with *[Osm]_LU_* that is not high, *[Na^+^]_U_* > 20 mmol/L [13,107] and may have hypervolemia, hypovolemia, or euvolemia.

Hypotonic hyponatremia typically associated with euvolemia is observed in glucocorticoid deficiency, hypothyroidism, high fluid intake accompanied by low solute intake (fluid intake during exercise, primary polydipsia, beer potomania, tea and toast diet, dilute infant formula), SIADH from various medical conditions and from drugs stimulating the synthesis or release of ADH [49], and conditions with features like SIADH [13,51]. The list of conditions causing SIADH is expanding [51,53,111]. The stimuli for ADH secretion include elevated *[Osm]_LS_* and serum tonicity, hypovolemia, and hypotension [112,113], but also pain, ADH production in tumors or in inflammatory clinical states, and from infused or ingested compounds. In SIADH, high levels of ADH cause water retention with expansion of both the intracellular and the extracellular volumes although the extracellular solutes are decreased due to high natriuresis, hypotonic hyponatremia, and high values of *[Osm]_LU_* and *[Na^+^]_U_* [114,115,116]. Excessive fluid intake may complicate the diagnosis of SIADH [117,118].

Two conditions have clinical and biochemical features like SIADH: In the nephrogenic syndrome of inappropriate antidiuresis, which is caused by X-linked mutations of the vasopressin receptor 2 (AVPR2), the serum ADH level is undetectable [119]. In the reset osmostat, the second of these conditions that occurs in various diseases, ADH is secreted at levels of *[Osm]_LS_* below the range that causes this secretion normally, e.g., at *[Osm]_LS_* < 280 mOsm/kg H_2_O [120]. This syndrome is manifested as chronic hypotonic hyponatremia resistant to routine treatments. It is differentiated from SIADH by the urinary diluting test or by newer tests, e.g., low fractional uric acid excretion [121].

Fluid losses from the kidney (e.g., from diuretics or mineralocorticoid deficiency), the gastrointestinal tract, the respiratory tract, or the skin, may cause hypovolemia, thirst, and high water intake resulting in hyponatremia [13,51]. Hyponatremia from renal losses is characterized by *[Na^+^]_U_* >20 mmol/L [13] or >30 mmol/L [51]. In hyponatremia from extrarenal losses, *[Na^+^]_U_* is <10 mmol/L [13] or <30 mmol/L [51]. *[Na^+^]_U_* may be changed from various influences, e.g., poor alimentation causing low *[Na^+^]_U_* values in SIADH [122], diuretics causing high *[Na^+^]_U_* values that create difficulties with the differentiation of SIADH from other causes of hyponatremia [123], or acid–base disturbances affecting renal sodium handling [124]. Gastrointestinal losses may cause hypovolemic hypotonic hyponatremia or hypernatremia [125].

### 2.5. Differentiating Between Euvolemic and Hypovolemic Hypotonic Hyponatremia

Clinical examination has poor sensitivity and specificity for differentiating euvolemia and hypovolemia [126]. The distinction between SIADH and cerebral salt wasting (CSW) exemplifies the diagnostic challenges in hyponatremias with normal or low *ECV*. These syndromes are characterized by elevated serum ADH, *[Osm]_U_*, and *[Na^+^]_U_*. Both CSW [127,128] and SIADH [129] complicate central nervous system diseases. In SIADH, ADH is secreted by tumors or in response to high interleukin-6 (IL-6) levels in inflammatory clinical states [111,130]. In CSW, high levels of the haptoglobin-related protein without signal peptide (HPRwsp), an acute-phase reactant, cause natriuresis, hypovolemia, and ADH secretion [111].

The difficulty of differentiating between CSW and SIADH in patients with neurological diseases by clinical examination [126,131,132] has led to the use of biochemistry measurements, including serum renin, aldosterone, natriuretic peptides, serum urea/creatinine ratio, urea and uric acid fractional excretion, and imaging techniques, e.g., the point-of-care ultrasound, which combines the degree of collapsibility of the inferior vena cava with cardiac and pulmonary ultrasonography [133]. The accuracy of several of these methods is also questionable [134].

The need to distinguish between SIADH and CSW was debated. One report proposed the differentiation of the two conditions by biomarkers or by infusion of isotonic saline, which corrects the high ADH secretion in CSW but not in SIADH [135]. A second report suggested that distinguishing the two conditions is unnecessary because they both are characterized by salt loss and should be treated by hypertonic saline infusion when symptomatic [136]. A third report stressed the need to study the function of biomarkers in CSW and the risk of worsening the hyponatremia in SIADH by increasing the natriuresis with saline infusion [137].

The categories of hyponatremia may also be differentiated by the ratio of the concentrations of calcium and creatinine in urine (*[Ca^++^]_U_/[Cr]_U_*). In one study, *[Ca^++^]_U_/[Cr]_U_* was 0.230 ± 0.096 in SIADH, with a significant decrease in this ratio after correction of the hyponatremia, 0.056 ± 0.038 in hypovolemic hyponatremia, and 0.034 ± 0.010 in hypervolemic hyponatremia [138].

Several biomarkers have been used to identify the volume category of hyponatremia. The natriuretic peptides used [139,140,141,142,143,144] have limited usefulness [134]. Fractional urate excretion [123,135,144,145,146,147] is particularly useful in differentiating between SIADH and CSW. This fractional excretion is high in both conditions at presentation and returns to within the normal range (3–11%) after correction of the hyponatremia in SIADH, but not in CSW [135]. High serum IL-6 is helpful in the diagnosis of SIADH from inflammatory conditions [111,131,148]. High levels of HPRwsp may establish the diagnosis of CSW [135] and are also observed in high renal losses causing hypotonic hyponatremias, e.g., in dementias [149,150,151,152,153].

ADH, copeptin, and neurophysin II are the components of the protein preprovasopressin in the posterior pituitary. Copeptin, which is studied in conditions in which evaluating blood ADH levels may be useful [154,155,156,157,158,159,160,161,162,163,164,165,166,167,168,169,170,171], is secreted from the pituitary in equimolar amounts to ADH [165]. Assay of ADH is unreliable [165]. Copeptin assay is dependable [167]. Copeptin levels may characterize various dysnatremias [155,156,157,158,159,163,166,167,168,169,170,171] or be useful in the diagnosis and prognostication of various diseases [161,162,163]. In hyponatremias, a copeptin/*[Na^+^]_U_* ratio ≤ 30 pmol/mmol assists in confirming the presence of euvolemia, and a serum copeptin < 3 pmol/L combined with *[Osm]_U_* < 200 mOsm/kg confirms the diagnosis of primary polydipsia [154].

**Figure 2:**
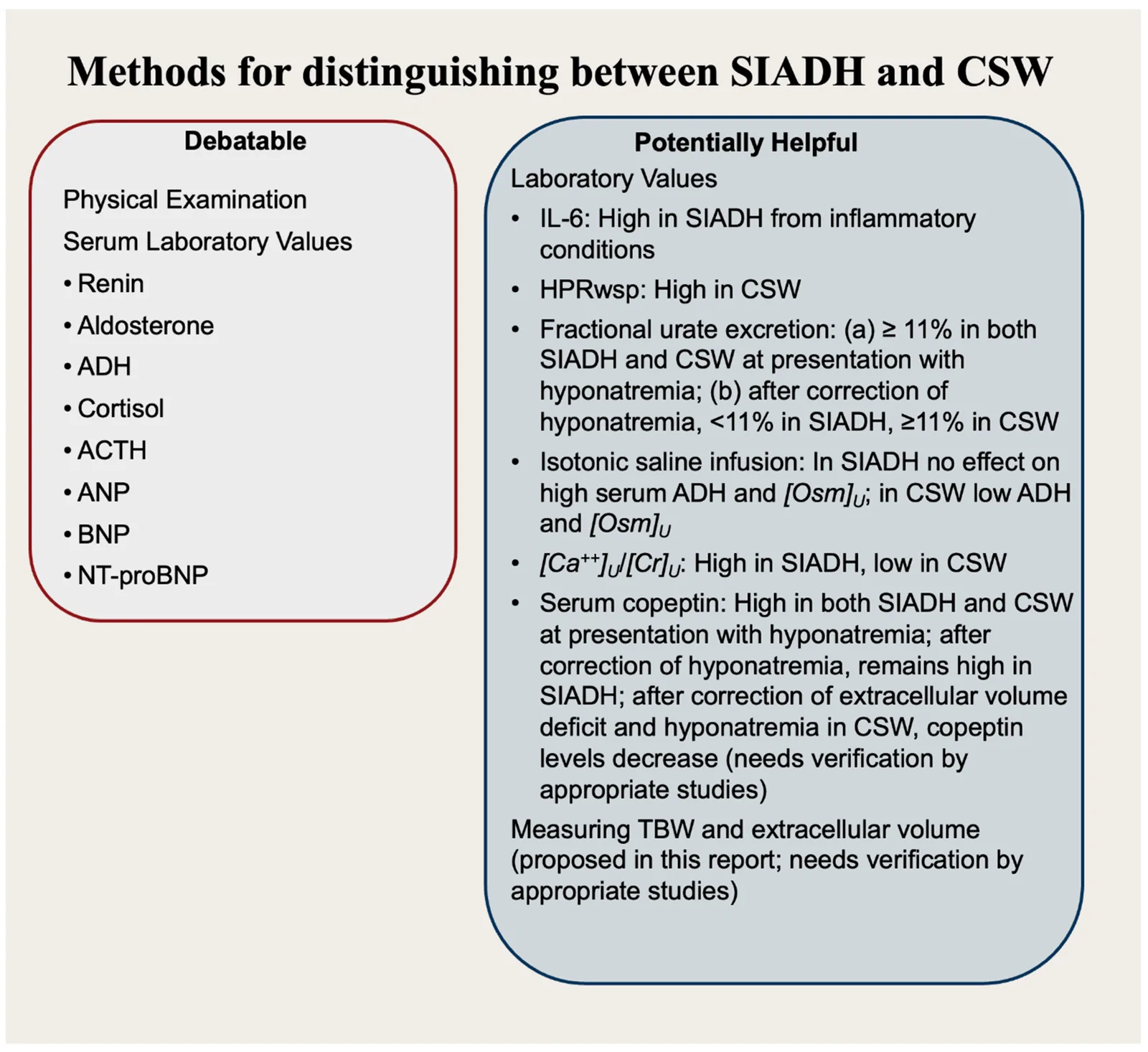
Distinguishing between SIADH and CSW.

The use of biomarkers in hyponatremias faces obstacles. Their measurements are not available in many hospital laboratories. When measurements are available, results are obtained with delays and their cost is high. An alternative method for assessing volume status in hyponatremia involves measuring *TBW* and *ECV* [140]. This method warrants further study. Figure 2 summarizes the methods for distinguishing between SIADH and CSW.

Prospective studies are needed using repeated measurements of biomarkers, especially of IL-6, HPRwsp, and copeptin, and repeated measurements of *TBW* and *ECV* at the bedside at presentation and during the correction of the hyponatremia.

**Figure 3:**
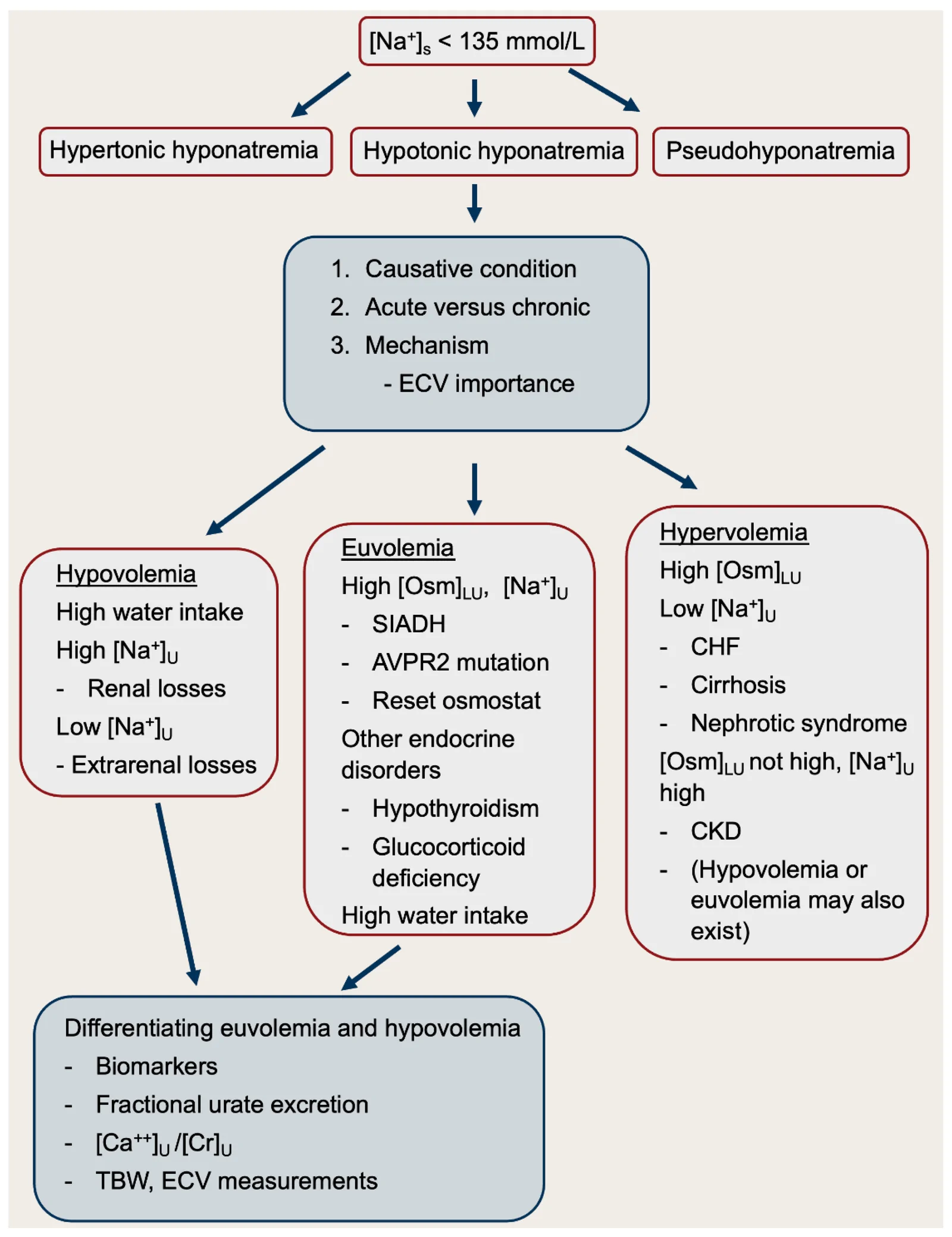
Diagnostic approach to hypotonic hyponatremia.

Figure 3 summarizes the diagnostic approach to hypotonic hyponatremia.

## 3. Treatment of Hypotonic Hyponatremia

The treatment of hypotonic hyponatremia aims to increase the fraction (*TBNa_Exch_* + *TBK_Ech_*)/*TBW* [4,5,39,40,41,42,43,44,45,46,47,48,49,50,51,52,53,54,55]. Patient survival, correction of clinical manifestations, and prevention of treatment-related complications are major treatment aims [5]. The treatment requires careful diagnostic evaluation and clinical and laboratory monitoring. The treatment methods are classified in this perspective according to the specific pathophysiological mechanisms they address and the general categories of hyponatremia for which they are indicated.

### 3.1. Treatment of the Medical Condition Responsible for the Hyponatremia

This approach is needed for all cases of hypotonic hyponatremia. The list of conditions that need to be evaluated and treated includes endocrine disorders (e.g., hypothyroidism or adrenal insufficiency), conditions causing SIADH or CSW, conditions causing hypervolemia or hypovolemia, etc.

### 3.2. Restricting Fluid Intake

Excessive fluid intake causes or worsens hypotonic hyponatremia and impedes the effectiveness of all the methods for raising *[Na^+^]_S_*. Restricting oral fluid intake is indicated for all categories of hyponatremia. Additional measures to fluid restriction are often required for many hypotonic hyponatremias, e.g., SIADH [4,5,42,54,172,173,174,175,176,177]. In hypovolemic hyponatremia, fluid restriction should be accompanied by saline infusion.

### 3.3. Methods for Increasing the Electrolyte-Free Urine Volume

These methods are indicated for conditions with high blood ADH levels and concentrated urine. The methods for increasing the electrolyte-free urine volume and for estimating the required volume of fluid restriction are based on the electrolyte-free water clearance ($C_{{eH}_{2}O}$):

$$C_{eH_{2}O}=\;V_{U}\times \left(1-\frac{{\left[Na^{+}\right]}_{U}\;+\;[K^{+}{]}_{U\;}}{[Na^{+}{]}_{S}}\right),$$

where *V_U_* is the urine volume over 24 h [178] and *[K^+^]_U_* is the concentration of potassium in the urine. Electrolyte-free water clearance is obtained at (*[Na^+^]_U_* + *[K^+^]_U_*)*/[Na^+^]_S_* < 1 [179]. Increasing free water excretion by decreasing this fraction and by increasing *V_U_* is obtained by osmotic diuresis, loop diuretics, and medications that block the ADH effect in the renal medulla.

Osmotic diuresis is produced by solutes excreted in urine [180,181,182,183,184] or by medications. Oral intake of salt and urea are the two methods with significant clinical experience for correcting hypotonic hyponatremia. Salt intake is not indicated in hypervolemic hyponatremia. Urea administration is effective, safe [185,186,187,188,189,190,191,192], and especially useful in cases of SIADH [185,186,187,188]. Its cost is low [185,190]. The daily doses of urea vary greatly [184,188]. A bitter taste is a drawback of urea intake. Formulations improving this taste are available [55,192]. Urea administration may not be indicated in hypovolemic hyponatremia or in CKD.

Osmotic diuresis from hypertonic mannitol infusion, which prevented hypotonic hyponatremia in rats infused with hypotonic saline [193], was proposed for the treatment of hyponatremia [194]. However, mannitol infusion may cause acute renal failure [195,196] and hypertonic hyponatremia, which complicates the evaluation of water and effective solute balances during the treatment of hypotonic hyponatremia [197].

Sodium-glucose co-transporter 2 (SGLT2) inhibitors cause glucosuria and osmotic diuresis. They have been evaluated in SIADH [198,199,200]. Various other hypotonic hyponatremias, e.g., hypervolemic hyponatremia in CHF, may also be managed using SGLT2 inhibitors [55].

Loop diuretics produce relatively higher excretion of water than sodium plus potassium [42,201]. Furosemide and bumetanide [202] have been used in hyponatremia. The urinary excretion of water and monovalent cations is similar at furosemide:bumetanide dose 40:1 in normal subjects [203] and in patients with CHF [204,205,206] or with nephrotic syndrome [207]. A ratio greater than 40:1 was required to obtain the same pattern or urinary electrolytes and water excretion in acute CHF [208]. Hypervolemic states constitute a clear indication for loop diuretics in hyponatremia [209]. In cirrhosis, guidelines request a combination of diuretics with other measures [210]. In SIADH, loop diuretics have been combined with isotonic saline to prevent hypovolemia [42]. However, a randomized controlled study in SIADH found no difference in the increase in *[Na^+^]_S_* among patients treated with fluid restriction alone, fluid restriction plus furosemide, or fluid restriction plus furosemide and saline [211]. Loop diuretics are contraindicated in hypovolemic hyponatremia.

Vasopressin receptor antagonists (vaptans) are used in euvolemic and hypervolemic hyponatremias with high blood ADH levels [212,213,214,215,216,217,218,219,220,221,222,223,224,225,226,227,228,229,230,231,232,233,234,235,236]. Currently, the appropriate uses of vaptans include hyponatremias not corrected by fluid restriction or other measures, SIADH, CHF, and non-severe hyponatremia. Their inappropriate uses include hypovolemic hyponatremias, hyponatremias treated with hypertonic saline infusion, history of hypersensitivity reaction to vaptans, severe loss of renal function, use of drugs that increase the blood levels of vaptans, poor thirst mechanism, liver disease, hyponatremias with low blood ADH, and transient high ADH states (cortisol deficiency, hypothyroidism, drug-induced SIADH) [237].

Lithium was used for correcting hyponatremia in SIADH [238]. It reduces the risk of severe hyponatremia in psychiatric patients treated with medications causing hyponatremia [239] and was proposed to be added to the treatment in these patients [240]. Like lithium, demeclocycline, which reduces the expression of aquaporin-2 in the renal medulla [241], increases electrolyte-free water clearance and was proposed for treating SIADH [242,243]. It was proposed that there is need for clinical and economic evidence for this use of demeclocycline [244]. With newer treatments, these two agents will probably not have much application in the future.

### 3.4. Method of Producing Concentrated Urine During Treatment of Hypotonic Hyponatremia

Infusion of desmopressin and saline was effective in safely correcting hyponatremia and reducing the incidence of overcorrection [245]. Desmopressin is indicated for hyponatremia with low ADH levels and *[Osm]_U_*. Hypovolemic hyponatremias, in which ADH secretion is suppressed after correction of hypovolemia, and hyponatremias due to excessive water intake or low solute intake are conditions in which this method is indicated [246].

Three strategies can be employed for desmopressin and saline infusion: (a) Proactive strategy in severe hyponatremia; (b) reactive strategy, based on changes in *[Na^+^]_S_* or urine output during treatment; and (c) rescue strategy, based on excessive increase in *[Na^+^]_S_* [247]. Overcorrection of hyponatremia by this strategy is managed by infusion of desmopressin and hypotonic fluids, e.g., 5% dextrose in water [248]. In proactive use, desmopressin and hypertonic saline should be infused together [247]. A study supported the reactive strategy [249]. In the rescue strategy, and to a lesser extent in the reactive strategy, patients are exposed to the risk of overcorrection. In a study of hypertonic saline infusion, the patient group with proactive desmopressin infusion had no significant adverse events and a lower rate of overcorrection of hyponatremia than the group that did not receive proactive desmopressin [250].

### 3.5. Method of Improving a Low Effective Arterial Blood Volume Causing Hypotonic Hyponatremia

Albumin infusion prevented and corrected hyponatremia in patients with cirrhosis [251,252]. However, the quality of the studies was low. Prospective studies are needed to establish the effectiveness and safety of this procedure.

### 3.6. Saline Infusion for Correction of Hypotonic Hyponatremia

The main indication for isotonic saline infusion for hyponatremia is in hypovolemia [4,5,253]. Hypertonic saline is indicated in hyponatremia with severe clinical manifestations [4,5], which consist of severe headache, vomiting, somnolence, seizures, noncardiogenic pulmonary edema, and hypoxia [4,5,44,254,255]. Specific targets for the rise of *[Na^+^]_S_* with saline infusion have been proposed [4,5,47,256]. Hypertonic saline is infused either continuously or in boluses [257]. These two methods have not shown significant differences in safety or effectiveness [258,259,260]. However, the rapid bolus method causes faster rise in *[Na^+^]_S_* and improvement in neurological manifestations and lower incidence of therapeutic relowering of *[Na^+^]_S_* than the continuous infusion [258,259]. The slow, continuous infusion is associated with shorter hospital stays [260].

Two methods have also been applied to determine the infused volume of hypertonic saline. One method consists of infusing the same volume of 3% saline (sodium concentration 513 mmol/L), e.g., 0.500 L [261], 0.150 L [4,262], or 0.100 L [263,264], to all patients. A fixed volume of hypertonic saline will achieve the prescribed rise in *[Na^+^]_S_* in subjects with only one value of *TBW* according to the Rose formula. In a study of infusion of the same bolus, final *[Na^+^]_S_* was below the target value in many patients with body weight > 100 kg and above the target value in many patients with body weight < 60 kg [265]. In another study, overcorrection of hyponatremia by >5 mmol/L was observed in 22.6% of cases after one bolus and in 34.6% after the second bolus [266]. One set of guidelines proposed infusing hypertonic saline at a volume in mL equal to the patient’s body weight in kg hourly to increase *[Na^+^]_S_* by 1 mmol/L in SIADH [4]. A second set of guidelines [5] proposed infusing 2 mL/kg of 3% saline or using the Adrogué–Madias formula [267] to compute the infused volume of hypertonic saline in patients with deviant body composition.

The second method for hypertonic saline infusion calculates the infused volume by formulas derived from the Edelman or Rose formulas. The components of the formula used early were the desired increase in *[Na^+^]_S_*, the sodium concentration in the infused saline, and a fixed estimate of *TBW*. Subsequently, several formulas have been proposed [201,267,268,269,270]. The Adrogué–Madias formula [267] added the volume of infused hypertonic saline to its components. Formulas for estimating the volume of infused saline should account for all the determinants of the change in *[Na^+^]_S_*. Both the formulas that do not account [201,267] and the formulas that account [268,269,270] for losses of water, sodium and potassium during treatment compute the volume of saline, plus the potassium salts added to the saline, that must be retained in the body to achieve the target rise in *[Na^+^]_S_*. This provides the rationale for monitoring *V_U_*, *[Na^+^]_U_*), and *[K^+^]_U_* during treatment [95,271].

The current formulas have limitations [272,273,274]. In addition to accounting for all determinants of the change in *[Na^+^]_S_* during infusion of hypertonic saline, a perfect formula should assign to each determinant the correct quantity [273]. The formulas lack determinants of the change in *[Na^+^]_S_*, e.g., sodium exchanges between the osmotically active and inactive compartments [77], and do not account or account poorly for water, sodium, and potassium losses or gains beyond the infused amounts during the treatment [273]. Excessively rapid correction occurs when renal water loss exceeds the losses of sodium and potassium during hypertonic saline infusion. In one study [275], excessive increases in *[Na^+^]_S_* were reported after infusion of hypertonic saline, the volume of which was calculated using the Adrogué–Madias formula [267]. These increases were primarily attributed to water diuresis [276].

Several studies reported reasonable accuracy of formulas computing the infused volume of saline [277,278,279,280,281,282]. Overcorrection was noted in hyponatremias with hypovolemia or caused by excessive water intake or solute intake [277]. A formula that included measured *V_U_* and *[Na^+^]_U_* during treatment yielded more accurate results than formulas assuming these values [278]. Lower incidences of under-correction and overcorrection were reported with hypertonic saline infusion using a formula than without a formula [281]. The proper use of any of the five formulas is not focused on the choice of the formula but on the selection of the proper *[Na^+^]_S_* change, the correct values of the determinants of this change entered in the formulas, and on monitoring of both the clinical status and serum and urine parameters during and after treatment [94,274,283,284,285,286,287]. Saline infusion can be effective and safe with proper monitoring [4,5].

Spurious *TBW* estimates entered in the formulas computing the infused volumes of saline cause errors [273]. A rapid, safe, easy-to-use, and accurate method for repeated bedside measurement of *TBW* would improve formula accuracy. Bioimpedance (BIA), proposed to estimate *TBW* in hyperglycemia with large fluid losses [288], may meet these requirements. BIA has been used in conditions with volume disturbances [289], including in differentiating between euvolemic and hypovolemic hyponatremia [140]. In dialysis patients, BIA was found to be reproducible and to have medium-to-high accuracy [290,291,292], but to underestimate tissue overhydration [293]. One advantage of BIA is its point-of-care applicability. Validation of BIA or another reliable method [294] for repeatedly measuring *TBW* and *ECV* during saline infusion is an important research target in the treatment of severe hyponatremia. Adding potassium infused and estimates of losses to the formulas has increased their complexity. Formula complexity has been criticized for its use in estimating hyperglycemic water and monovalent cation deficits [295]. Complex formulas and point-of-care frequent measurements of *TBW*, *ECV*, serum and urine parameters must result in improved outcomes of treatment of hyponatremia to justify their use. Prospective studies addressing these treatment methods are needed.

### 3.7. Treatment of Hypotonic Hyponatremia by Dialytic Methods

Hyponatremia has been corrected by continuous renal replacement therapy (CRRT) [296,297], hemodialysis [298,299], and peritoneal dialysis [300,301]. High *[Urea]_S_* levels were associated with reduced risk of neurological complications during rapid correction of hyponatremia via hemodialysis [302,303]. Correction of hyponatremia by hemodialysis produces a set of brain conditions that differ from those present in the absence of azotemia. A beneficial effect of azotemia, observed in rats, is the rapid accumulation of organic osmolytes following correction of chronic hyponatremia [304]. However, osmotic demyelination syndrome (ODS) developed during the first hemodialysis session of a patient with rapid correction of hyponatremia and a rapid decrease in *[Urea]_S_* [305]. During a vigorous hemodialysis session, the exit rate of urea from brain cells is slower than the rate of decrease in *[Urea]_S_*, resulting in osmotic swelling of brain cells [306] and in the hemodialysis disequilibrium syndrome [307]. The risk of ODS, which may not be present in patients on chronic hemodialysis, is present when acute hemodialysis is used to correct hyponatremia [308]. In this case, slow hemodialysis is required to balance the rate of urea removal with the relatively high sodium concentration in the dialysis fluid [309]. Development of point-of-care methods for monitoring *[Na^+^]_S_* and modifying the composition of the dialysis fluid during treatment would be beneficial [310].

**Figure 4:**
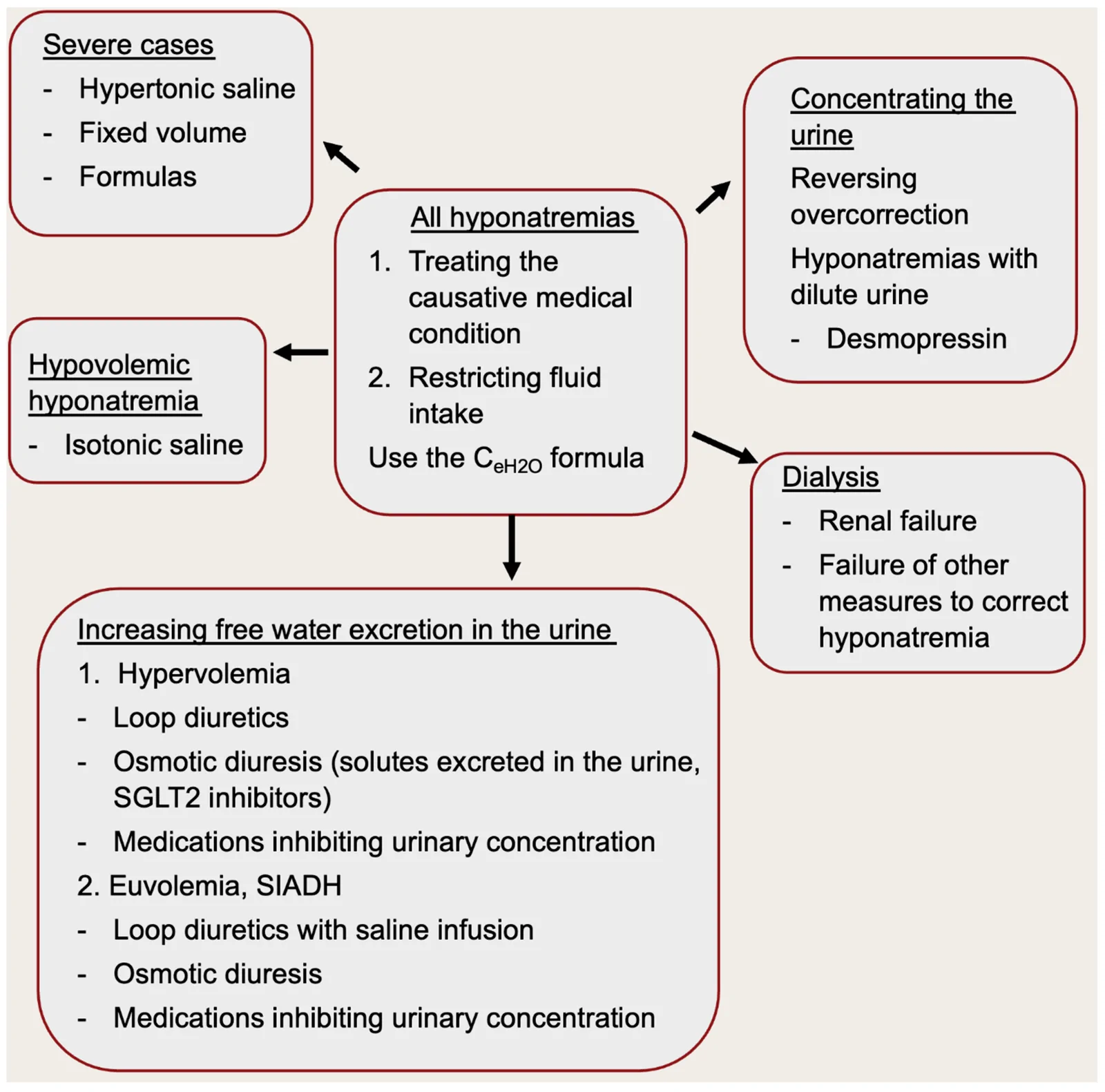
Treatment of hypotonic hyponatremia.

Figure 4 presents the indications for using various methods for correcting hypotonic hyponatremia.

## 4. Areas of Uncertainty and Controversy in the Management of Hyponatremia

The methods of treating hypotonic hyponatremia must prevent the complications of treatment, including adverse effects of medications used, symptomatic hypervolemia from saline infusion, and prevention of neurologic complications. ODS as a complication of hyponatremia treatment has received attention recently.

### 4.1. Prevention of Osmotic Demyelination Syndrome: Rate of Correction of Hyponatremia

Rapid rate of correction of hypotonic hyponatremia was reported to be a risk factor for development of ODS [51,311,312,313,314,315,316,317,318]. The risk factors include also *[Na^+^]_S_* < 120 mmol/L, chronic hyponatremia, alcoholism, severe liver disease, liver transplant, malnutrition, hypokalemia, hypophosphatemia, and hypochloremia [319,320,321,322,323]. ODS has developed in patients with these conditions and without hyponatremia [322]. In guidelines, the limits of increase in *[Na^+^]_S_* are 10 mmol/L over 24 h and 18 mmol/L over 48 h for patients with chronic hyponatremia [324], with initial rapid rise of 4–6 mmol/L to correct severe symptoms of hyponatremia [4], and 5–10 mmol/L in the first 24 h, and then 8 mmol/L daily for both acute and chronic hyponatremias [5]. Various other rates of correction have been proposed [325,326,327]. A rate of correction up to 8 mmol/L per 24 h has been considered appropriate.

An early small study disputed the notion that rapid correction of hyponatremia constitutes a risk for ODS [328]. Recent large studies could be interpreted as supporting this notion and faster correction. In one study, ODS was diagnosed in small fractions of patients with both slow and fast hyponatremia correction [329]. In another study, slow correction rate was associated with higher mortality and longer hospitalization, and that the role of the correction rate in neurologic complications needs further studying [330]. In a third study, the incidence of death and neurological complications were higher at hyponatremia correction rates < 8 mmol/L per 24 h than at higher correction rates [331]. One review concluded that rapid correction of hyponatremia has a survival benefit, and slow correction is associated with increased risks of mortality and length of hospitalization [332]. Three other reviews concluded that rapid correction is associated with ODS, but ODS develops also in patients with slow correction [333,334,335].

The appropriate rate of correction of hyponatremia is currently debated [336]. One group proposes rate of correction > 8 mmol/L in the first 24 h and up to 15 or 20 mmol/L over 48 h [337,338]. A second group, which supports the slower correction rate [339,340], stresses the need for a clear definition of ODS, which had not been followed in all the studies [340]. The observational studies analyzing the relation between observed hyponatremia correction rates and clinical outcomes have potential deficits [341]. The rate of correction of hyponatremia associated with risk factors, particularly with chronicity and very low *[Na^+^]_S_* values, needs to be studied by prospective randomized methods [341,342].

### 4.2. Prevention of Osmotic Demyelination Syndrome: Medications

Dexamethasone [343], and minocycline [344] prevented ODS in animals with chronic hyponatremia receiving infusions of hypertonic saline. Prospective studies are needed comparing patients with hyponatremia treated with hypertonic saline and these drugs to those treated only with hypertonic saline. One potential effect of urea administration consists of the protection of ODS [345]. In rats, rapid correction with intravenous urea of similar levels of severe hyponatremia induced by desmopressin and free access to water was associated with lower mortality and lower frequency of ODS than correction with hypertonic saline or vaptans [346]. Prospective studies on parenteral urea administration to protect ODS during treatment of severe hyponatremia are needed.

Finally, potassium replacement could be studied for prevention of ODS. Loss of intracellular potassium, along with sodium and chloride, occurs early in hyponatremia. In rats with severe hyponatremia induced by continuous desmopressin infusion, the loss of potassium from brain cells exceeded the loss of sodium [347]. However, potassium replacement was associated with the development of ODS in a patient with severe hyponatremia [348]. Whether potassium replacement during treatment of hyponatremia with normal serum potassium mitigates the risk of ODS is unclear. Animal studies are needed to address this hypothesis. Potential methods of protecting ODS in hyponatremia should be studied in patients with carefully constructed protocols and extreme caution.

### 4.3. Other Hyponatremia Management Methods: Research Requirements in Hyponatremia

Patients who require detailed diagnostic workup and monitoring during the correction of *[Na^+^]_S_* require hospitalization [5]. The ability of the treatment team to identify the mechanism of hyponatremia and use the proper method of treatment is critical. In one study, the pathogenesis of hyponatremia was not evaluated appropriately in a large fraction of hospitalized patients, and an even larger patient fraction remained hyponatremic at discharge [349]. In a randomized study of hospitalized patients with chronic hyponatremia [173], the results of hyponatremia correction were compared between a patient group treated by a specialized team using a protocol and a group treated by physicians using the European guidelines [5]. In both groups, less than 10% of the patients had severe hyponatremia. The treatment produced a higher rate of normonatremia in the group treated by the specialized team, but the rates of 30-day mortality and rehospitalization did not differ between the two groups. Death and rehospitalization within 30 days were lower in patients who had normonatremia at discharge in both treatment groups.

Patients with severe hyponatremia require intense diagnostic approach and treatment methods. They should be admitted to the intensive care unit and managed using a carefully constructed diagnosis and treatment protocol. Treatment methods requiring intensive care medicine include saline infusion, desmopressin infusion, specific treatments to prevent ODS, and combination therapies. The treatment protocol should contain in detail the method of selecting patients for infusion of saline or desmopressin, the method of saline infusion, the desired rate(s) of rise of *[Na^+^]_S_*, and the type and frequency of monitoring blood and urine tests, which ideally should be monitored by point-of-care methods. For all patients treated for hyponatremia, the patients and their families should receive careful instructions about fluid and solute intake, and the patients primary care providers should review medications used that may cause dysnatremias.

Overall, evidence exists of substantial improvement in mortality from severe hyponatremia when it is treated aggressively by dedicated specialists [350]. However, there is a lack of robust randomized controlled treatment trials [34]. Table 4 presents diagnostic and treatment areas of hypotonic hyponatremia that require further investigation, identified in this perspective.

## 5. Conclusions

The diagnosis and treatment of hypotonic hyponatremia have complexities that are still strongly debated. Carefully planned and conducted prospective studies are needed to address these issues and improve management outcomes. Biomarkers and bedside measurements of *TBW* and *ECV* should be studied for establishing the volume status. Severe hyponatremia should be managed in hospitals. The hyponatremia should be corrected during hospitalization. A rapid rate of correction should be planned for acute hyponatremia. Major therapeutic topics requiring research include the comparison of methods for correction of hyponatremia combined with fluid restriction, improving the formulas computing the volume of hypertonic saline needed for specific rises in *[Na^+^]_S_*, and improving the methods of prevention of ODS by studying the rate of correction of *[Na^+^]_S_* in conditions predisposing to ODS and by pharmacological interventions.

## Figures and Tables

**Table 1:** Parameters of serum tonicity.

Tonicity Index	Definition
*[Na^+^]_S_*, mmol/L	Sodium concentration in serum
*[Osm]_LS_,* mOsm/kg H_2_O	Total solute concentration in serum water. *[Osm]_LS_* is measured by a colligative property, usually depression of the freezing point of the serum.
*[Osm]_RS_*, mOsm/L	Total solute concentration in serum, not in serum water. *[Osm]_RS_* is not measured. It is computed by formulas. Frequently used formula: *[Osm]_RS_* = $2\times \;\;\left[Na+\right]s+\left[Glucose\right]s+\left[Urea\right]s$
*[Gap]_OsmS_*, mOsm/L	Difference between *[Osm]_LS_* and *[Osm]_RS_*: *[Gap]_OsmS_* = *[Osm]_LS_* − *[Osm]_RS_*
*[Ton]_S,_* mOsm/L	Total concentration in serum of solutes with extracellular distribution. Serum tonicity is computed by the formula: *[Ton]_S_* = $2\times \left[Na^{+}\right]s+\left[Glucose\right]s$ This formula is specific for hyperglycemia
*[Na^+^]_SW_,* mmol/L	Sodium concentration in serum water

*[Glucose]_S_* = serum glucose concentration_,_ mmol/L; *[Urea]_S_* = serum urea concentration, mmol/L.

**Table 2:** Classification of pure forms of hyponatremias according to tonicity category.

Hyponatremia						
Category	*[Na^+^]_S_*	*[Osm]_LS_*	*[Osm]_RS_*	*[Gap]_OsmS_*	*[Ton]_S_*	*[Na^+^]_SW_*
**Hypotonic**	Low	Low	Low	Normal	Low	Low
**Hypertonic:**						
**Hyperglycemia**	Low	High	High	Normal	High	Low
**Hypertonic:**						
**Exogenous Solute**	Low	High	Low	High	Low	Low
**Pseudohyponatremia**	Low	Normal	Low	High	Low	Normal

*[Osm]_RS_*, *[Gap]_OsmS_*, and *[Ton]_S_* in this Table are values calculated by the formulas presented in Table 1.

**Table 3:** Formulas determining *[Na^+^]_SW_* and *[Na^+^]_S_*.

**Reference**	Formula
Edelman, I.S.; et al. [73]	$\left[Na^{+}\right]sw=1.11\times \frac{TBNa_{Exch}+TBK_{Exch}}{TBW}-25.6$
Rose, B.D. [75]	$\left[Na^{+}\right]s=\frac{TBNa_{Exch}+TBK_{Exch}}{TBW}$

*TBNa_Exch_*, mmol; *TBK_Exch_*, mmol; *TBW*, L.

**Table 4:** Topics of hypotonic hyponatremia requiring investigation.

Topic to be Investigated	Proposed Methods
**Diagnosis topics**	
-Establishing the diagnosis of SIADH combined with other hydration disturbances	-Assessing a mathematical model [118]
-New methods for diagnosing reset osmostat	-Fractional excretion of urate <11%
-Differentiating SIADH from CSW	-Methods presented in Figure 2
-Study of biomarkers in differentiating various categories of hyponatremias	-Prospective studies of IL-6, HPRwsp and copeptin
**Treatment topics**	
-Safety and efficacy of the methods needed for correcting hyponatremia in addition to restricting fluid intake	-Randomized controlled trials of urea, SGLT2 inhibitors, loop diuretics, vaptans, and other measures
-Efficacy and safety of methods for desmopressin infusion	-Studies comparing the various methods
-Efficacy and safety of albumin infusion in patients with hepatic cirrhosis	-Prospective studies needed
-Accuracy, efficacy and safety of computing by formulas the volume of infused hypertonic saline for a prescribed rise in *[Na^+^]_S_*	-Prospective studies of reported formulas using *TBW* values obtained by BIA
-Improving the formulas by studying factors not included in the Edelman study and by measuring *TBW*	-Studies in animals and in humans with hyponatremia of change in *[Na^+^]_S_* produced by hypertonic saline infusion computed by formulas
-Establishing optimal rates of increase in *[Na+]_S_* in various patient categories	-Randomized controlled trials comparing fast and slow rates of correction of hyponatremia in patients with and without conditions identified as predisposing to ODS
-Safety and efficacy of pharmacological methods for prevention of ODS	-Prospective studies
-Safety and efficacy of dialytic methods for the management of hyponatremia	-Prospective studies

## Data Availability

No new data were created or analyzed in this report. Data sharing is not applicable to this study.

## Abbreviations

**Abbreviation**	**Abbreviated Entity**
*[Na^+^]_S_*	Serum sodium concentration
*[Na^+^]_SW_*	Sodium concentration in serum water
*[Na^+^]_Scor_*	Serum sodium concentration in hyperglycemia corrected for normalization of serum glucose without changes in body sodium, potassium, or water
*[Na^+^]_U_*	Sodium concentration in urine
*TBNa_Exch_*	Total body exchangeable sodium
*[K^+^]_U_*	Potassium concentration in urine
*V_U_*	Urine flow rate
*TBK_Exch_*	Total body exchangeable potassium
*TBW*	Total body water
*SWC*	Serum water content
*SSC*	Serum solid content
*[Osm]_LS_*	Serum osmolality
*[Osm]_RS_*	Serum osmolarity
*[Gap]_OsmS_*	Serum osmol gap
*[Osm]_LU_*	Urine osmolality
*[Ton]_S_*	Serum tonicity (effective osmolarity)
CHF	Congestive heart failure
CKD	Chronic kidney disease
ADH	Antidiuretic hormone (vasopressin)
SIADH	Syndrome of inappropriate antidiuretic hormone secretion
*ECV*	Extracellular volume
*EABV*	Effective arterial blood volume
AVPR2	Arginine vasopressin receptor 2
CSW	Cerebral salt wasting
Il-6	Interleukin-6
HPRwsp	Haptoglobin-related protein without signal peptide
ANP	Atrial natriuretic peptide
BNP	Brain natriuretic peptide
NT--proBNP	N-terminal pro b-type natriuretic peptide
ACTH	Adrenocorticotropic hormone
*[Ca^++^]_U_*/*[Cr]_U_*	(Urine calcium concentration)/(Urine creatinine concentration)
BIA	Bioelectrical impedance
SGLT2	Sodium-glucose co-transporter 2
CRRT	Continuous Renal Replacement therapy
ODS	Osmotic demyelination syndrome
